# Anaesthesiologist: The ‘Best Man’ for the Robotic Patient

**DOI:** 10.21315/mjms2021.28.1.16

**Published:** 2021-02-24

**Authors:** Claudio Valotto, Fabrizio Dal Moro

**Affiliations:** 1 Department of Medical Area — Urology, University Hospital of Udine, Udine, Italy; 2 Department of Surgery, Oncology and Gastroenterology — Urology, School of Medicine and Surgery, University of Padova, Padova, Italy

Dear Editor,

We would like to congratulate Kapur and Kapur (1) for their excellent work on the effects of robot-assisted surgery on anaesthetic management. The authors performed a systematic review on the development of surgical robots, underlining the anaesthetic concerns.

They analysed the characteristics and limitations of robotic surgery, focusing on the anaesthesiologist’s point of view: in particular, they reported that epidural catheter placement helps with intra- and post-operative pain relief. Indeed, in several reported experiences about radical prostatectomy (2), the use of combined general and epidural anaesthesia determines a lower consumption of opioids during this minimally invasive procedure, with a consequentially lower incidence of post-operative nausea and vomiting (PONV) when compared with other experiences using general anaesthesia.

In our experience (3), we have obtained the same beneficial results performing a transversus abdominis plane-block (TAP-block): this procedure, performed after the induction of general anaesthesia, allows a significant reduction in intra- and post-operative use of analgesics, also resulting in a decrease in PONV. The mechanism seems to be the same as the one reported when using the epidural analgesia, confirming that a reduction in administered opioids is crucial for avoiding gastrointestinal disorders.

As reported by the above authors, there are other anaesthetic factors to be considered, such as the impact of robotic surgery on cardiopulmonary complications. Specifically, regarding this parameter, one of the most influential factors is represented by the approach. Indeed, the decision to use the extraperitoneal route in approaching the surgical field (i.e. during radical prostatectomy) is burdened by a greater risk of having a partial CO_2_ pressure significantly higher in comparison with the transperitoneal approach (4).

Robotic surgery is currently one of the most popular minimally invasive techniques, allowing shorter length of stay, lower blood loss and the possibility of better functional outcomes in comparison with other methods. Nevertheless, we need to keep in mind that this approach could also present dangers because there are many factors which need to be taken into account when selecting the best strategy for the patient.
